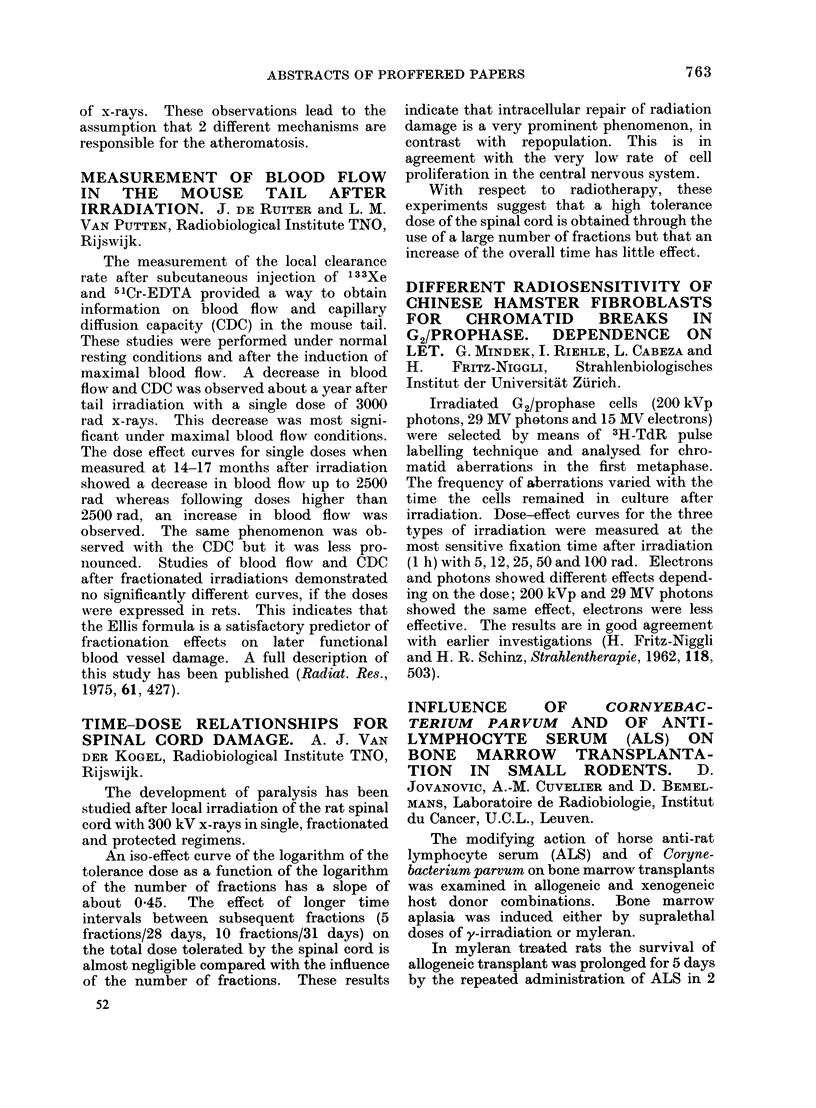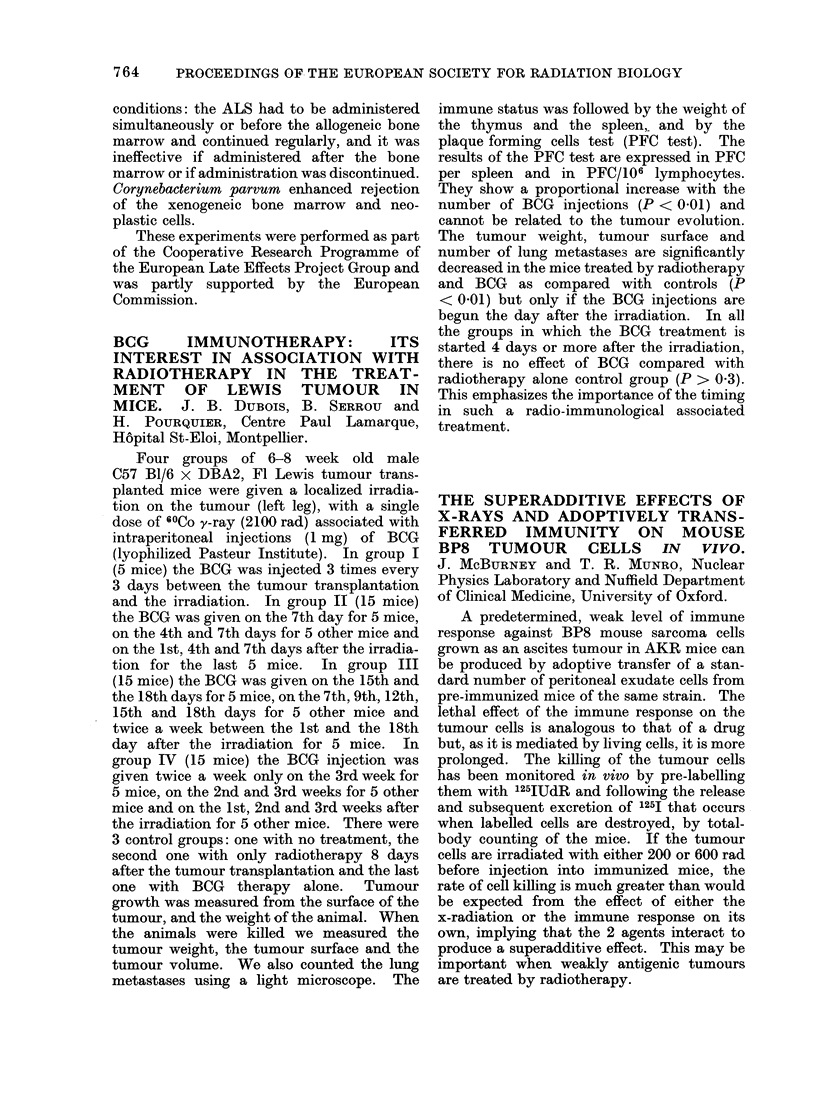# Proceedings: Influence of Cornyebacterium parvum and of anti-lymphocyte serum (ALS) on bone marrow transplantation in small rodents.

**DOI:** 10.1038/bjc.1975.331

**Published:** 1975-12

**Authors:** D. Jovanovic, A. M. Cuvelier, D. Bemelmans


**INFLUENCE OF *CORNYEBACTERIUM PARVUM* AND OF ANTI-LYMPHOCYTE SERUM (ALS) ON BONE MARROW TRANSPLANTATION IN SMALL RODENTS.** D. Jovanovic, A.-M. Cuvelier and D. Bemelmans, Laboratoire de Radiobiologie, Institut du Cancer, U.C.L., Leuven.

The modifying action of horse anti-rat lymphocyte serum (ALS) and of *Corynebacterium parvum* on bone marrow transplants was examined in allogeneic and xenogeneic host donor combinations. Bone marrow aplasia was induced either by supralethal doses of $\gamma$-irradiation or myleran.

In myleran treated rats the survival of allogeneic transplant was prolonged for 5 days by the repeated administration of ALS in 2

conditions: the ALS had to be administered simultaneously or before the allogeneic bone marrow and continued regularly, and it was ineffective if administered after the bone marrow or if administration was discontinued. *Corynebacterium parvum* enhanced rejection of the xenogeneic bone marrow and neoplastic cells.

These experiments were performed as part of the Cooperative Research Programme of the European Late Effects Project Group and was partly supported by the European Commission.